# LB1577. Outcomes of Pregnant Women Exposed to Sotrovimab for the Treatment of COVID-19 in the Omicron Predominant Era (PRESTO)

**DOI:** 10.1093/ofid/ofac492.1887

**Published:** 2022-12-15

**Authors:** Jessica Tuan, Manas Sharma, Jehanzeb Kayani, Matthew W Davis, Dayna McManus, Jeffrey E Topal, Onyema Ogbuagu

**Affiliations:** Yale University School of Medicine, New Haven, CT; Yale University School of Medicine, New Haven, CT; Yale University School of Medicine, New Haven, CT; Yale New Haven Hospital, New Haven, Connecticut; Yale New Haven Hospital, New Haven, Connecticut; Yale New Haven HospitalYale University School of Medicine,, New Haven, Connecticut; Yale School of Medicine, New Haven, Connecticut

## Abstract

**Background:**

Sotrovimab, a monoclonal antibody with efficacy against SARS-CoV-2 including certain Omicron variants, has been used in treatment of mild-moderate COVID-19. Limited data exists regarding its use in pregnant women.

**Methods:**

Electronic medical record review of pregnant COVID-19 patients treated with sotrovimab from 12/30/21-1/31/22 (Yale New Haven Health Hospital System [YNHHS]) was performed. Included were pregnant individuals ≥ 12 years, weighing ≥ 40 kg, with positive SARS-CoV-2 test (within 10 days). Those receiving care outside YNHHS or receiving other SARS-CoV-2 treatment were excluded. We assessed demographics, medical history, and Monoclonal Antibody Screening Score (MASS). Clinical outcomes assessed included emergency department (ED) visit < 24 hours, hospitalization, ICU admission, and/or death within 29 days of sotrovimab. Pregnancy and neonatal outcomes were assessed until 8/15/22.

**Results:**

Among 22 subjects, median age was 32 years and body mass index was 27 kg/m^2^. Sixty-three percent were Caucasian, 9% Hispanic, 14% African-American, and 9% Asian. Nine percent had diabetes and sickle cell disease. Five percent had well-controlled HIV. Eighteen percent, 46%, and 36% received sotrovimab in trimester 1, 2, and 3, respectively. No infusion/allergic reactions occurred. MASS values were < 4. Only 12/22 (55%) received complete primary vaccination (46% mRNA-1273; 46% BNT162b2; 8% JNJ-78436735); none received a booster.

There were no ICU admissions nor deaths. One subject was hospitalized for post-partum pyelonephritis; another had an ED visit for post-partum vaginal bleeding. Median gestational age at birth was 38.9 weeks. Nine percent had premature labor and premature rupture of membranes, respectively. Median infant birth weight was 3220 g. One neonate required an ICU stay due to prenatally diagnosed omphalocele (before sotrovimab) in a mother with congenital defect history. There were no abortions, fetal loss, or other birth/neurodevelopmental defects.

**Conclusion:**

Pregnant COVID-19 patients receiving sotrovimab at our center tolerated it well with good clinical outcomes. Pregnancy and neonatal complications did not appear sotrovimab-related. Though a limited sample, our data helps elucidate the safety and tolerability of sotrovimab in pregnant women.

**Disclosures:**

**All Authors**: No reported disclosures.

